# Viewpoint: Community-led social prescribing in low and middle income countries: a case study from Lao People’s Democratic Republic

**DOI:** 10.1016/j.lanwpc.2025.101681

**Published:** 2026-01-15

**Authors:** Shogo Kubota, Elizabeth M. Elliott, Ketkesone Phrasisombath, Phonpaseuth Ounaphom, Sengaloun Nhotleuxay, Ounkham Souksavanh, Khanphoungeune Volaot, Somchit Tanhtabud, Samkham Meunsy, Sandra Bode, Yu Lee Park, Timothy Armstrong

**Affiliations:** a World Health Organization, Country Office for Lao People's Democratic Republic, 125 Saphanthong Road, Unit 5 Ban Saphangthongtai, Sisattanak District, Vientiane Capital, Lao People's Democratic Republic; b Maternal Child Health and Quality Safety, World Health Organization Regional Office for the Western Pacific, P.O. Box 2932, Manila 1000, Philippines; c Department of Hygiene and Health Promotion, Ministry of Health, Vientiane, Lao People's Democratic Republic; d Department of Local Authorities, Ministry of Home Affairs, Vientiane, Lao People's Democratic Republic; e TREE+ for Sustainable Development, Vientiane, Lao People's Democratic Republic

**Keywords:** Social prescribing, Low and middle-income countries, Trust, Social determinants of health, Community engagement

## Abstract

Social prescribing has been mainly developed in high-income countries with strong health systems, while its application in low- and middle-income countries is less explored. In Lao PDR, the CONNECT Initiative offers context-specific support to improve community health and wellbeing through a bottom-up, participatory approach. Community members identify vulnerable groups and needs, then work together with local authorities and health staff to find solutions like improved water access, transport to health facilities, and support for the elderly. An evaluation indicates increased trust in the health system and uptake of essential services. This demonstrates how communities in low-resource settings can collectively develop solutions to address local social determinants of health, and the importance of building trust and addressing power imbalances. This community-led process in which health systems play a supporting role can be reframed as co-production rather than prescribing and may also provide insights for high-income countries in strengthening social cohesion.

## Background

Extensive research consistently demonstrates the profound impact of social determinants of health (SDH), with non-medical factors reported to account for over 80% of population health outcomes.^1^^,^^2^ SDH cannot be addressed through purely medical interventions; social prescribing has therefore been developed as a means of targeting the practical, social and emotional needs that affect health and wellbeing. In a recent global definition, social prescribing is defined as “a means for trusted individuals in clinical and community settings to identify that a person has non-medical, health-related social needs and to subsequently connect them to non-clinical supports and services within the community by co-producing a social prescription—a non-medical prescription, to improve health and wellbeing and to strengthen community collaborations”.^3^

Social prescribing has primarily been developed and utilised within high-income contexts. The first health system to introduce it was the NHS in the UK, and it has since been implemented in various forms in Europe, Asia and North America.^4^^,^^5^ High-income countries usually (but not always) possess common characteristics that might be prerequisite to the feasibility and effectiveness of social prescribing, including:

i) Relatively strong and accessible health systems, which have the capacity to identify the social needs of patients;
ii) Relatively strong established systems and referral pathways to respond to the identified needs beyond the health sector.^6^

In practice, there is considerable variation in approaches and little guidance on how social prescribing could be developed to reflect local health systems and needs.^7^ While overall evidence on the health impact of social prescribing has been inconclusive, there is particularly limited evidence of its application and adaption in Low and Middle Income Countries (LMICs) without robust health and social systems.^8^, ^9^, ^10^ Many LMICs have traditions of community health initiatives and grassroots action but these are rarely categorised as formalised social prescribing.^11^

The Lao People’s Democratic Republic (Lao PDR) has a predominantly rural population of 7.5 million and has been classified as a lower-middle income country since 2011. While the health system is still weak, the Ministry of Health has launched reforms to provide better services and work closely with development partners. Health care financing has heavily relied on external funding, so transition plans have been prioritized to respond to challenges created by the recent economic downturn and rapid inflation.^12^ However, despite a gradual increase in domestic expenditure on health and the introduction of various social health insurance schemes, the cost of reaching and using health services remains high and many people, especially the elderly, still experience catastrophic health expenditure.^13^ Barriers to accessing government health services include physical distance to healthcare facilities, lack of health information, bureaucratic procedures, language, judgemental attitudes of providers, and inconsistent provision of services,^14^ and access is impacted by socioeconomic disparities.^15^ Services such as mental health care are rarely available or utilised (an estimated 95% of psychological disorders go untreated),^16^ and counselling is often lacking or inadequate during points of contact such as antenatal care.^17^ Even if cases requiring social support are detected, there are limited or difficult to access formal referral mechanisms, and often poor trust in these systems; for example, only 3% of women experiencing gender-based violence seek support from health providers.^18^ There is therefore little capacity within the formal health sector to detect and respond to social needs. However, the governance system of Lao PDR (a single-party socialist state with strong links between central and subnational government down to the village level), and the relatively coherent village structure found in many places (including village chiefs, traditional ethnic leaders and representatives of mass organizations) means that community resources can be utilized.

The government-led CONNECT Initiative provides bottom-up, locally contextualized support for community engagement and local governance to improve community health and wellbeing in Lao PDR. Similarly to social prescribing in high-income countries, it includes actions to address SDH through community activities to meet social needs. However, unlike high-income settings, it does not rely on formal providers such as health or social services to diagnose issues and prescribe solutions. Instead, it supports communities to ‘self-diagnose’ and ‘self-prescribe’ social interventions through participatory village planning with health and government staff in a supporting role. In this viewpoint, we propose an expanded definition of social prescribing in low-resource settings where health sector-led prescribing may not be the most feasible or appropriate method, towards a model of community-led social prescribing or co-production. This viewpoint briefly details (1) the design of the initiative and its rationale; (2) evidence and findings to date as outcomes of the initiative, (3) examples of community-led social prescribing; (4) lessons learned from the initiative and the opportunities and challenges of social prescribing in low resource settings.

## The CONNECT initiative

In Lao PDR, the Ministries of Health and Home Affairs, with the support of WHO and partners, co-developed the CONNECT (Community Network Engagement for Essential Healthcare and COVID-19 Responses through Trust) Initiative. CONNECT aims to improve health equity through enhancing existing governance and community structures and capacities for overall community health and wellbeing, whilst also strengthening trust between villagers, health services and local government. The process was initiated during the COVID-19 pandemic in 2020, and has since led to a memorandum of understanding (MoU) between the Ministers of Health and Home Affairs and attracted various funding support from partners for nation-wide rollout. It supports community representatives, health and local government staff to build relationships and identify locally specific SDH, towards developing collaborative solutions which build on existing local resources. CONNECT does not adopt a top-down, prescriptive approach, but instead, supports communities to self-identify their needs as well as find resources to address those needs.

**Fig. 1:**
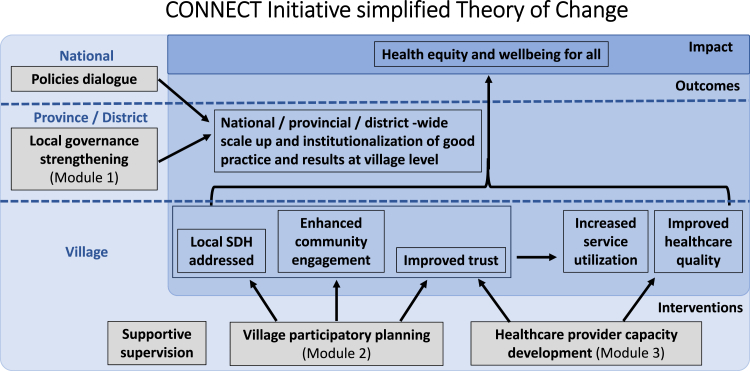
CONNECT initiative theory of change (simplified).

CONNECT includes three key modules which take the form of participatory workshops and community-based activities targeting different stakeholders and levels, which were developed over time through iterative and participatory processes over the initial phase of the initiative in 2020–2022.^19^ These aim to 1) strengthen local governance by gaining commitment from provincial and district governors and relevant multisector stakeholders to support and coordinate community engagement; 2) engage community representatives, beneficiaries, health staff and district officials to build trust and support villagers to identify vulnerable populations, community priorities and existing resources and jointly develop a plan to improve community health; 3) develop the capacity of healthcare providers to provide respectful care to gain trust from villagers and better respond to their needs towards increased essential health service use. The modules are followed by regular supportive supervision by central, provincial and district officials to institutionalize and integrate the approach into routine work and continue building trust. As shown in a simplified Theory of Change (Fig. 1) (discussed in more detail elsewhere^19^), this is combined with policies dialogue at national level towards the overall goal of developing health equity and wellbeing for all.

A mixed-method evaluation was jointly developed to assess the short-term outcomes of CONNECT. A village census survey (interviewing everyone above sixteen years old in selected villages before and after implementing CONNECT modules) showed a broad increase in trust through locally contextualised measurements and mild association with service uptake,^20^ and increased sense of ownership and responsibility for community health. Operational research and case studies have shown examples of increased service uptake especially in COVID-19 vaccination,^21^ and maternal care such as antenatal care and deliveries in health centres.^22^ Examples of scale-up by district authorities with domestic funds are reported through supportive supervision, which shows evidence of scalability through local governance although the findings are anecdotal. At a national level, the latest social indicator survey shows a trend for increased equity in healthcare access since 2017 although this cannot be attributed to CONNECT alone.^23^

## Community-led social prescribing

A key part of CONNECT is for community representatives to identify local SDH and support the development of community-led solutions through participatory planning. This was assessed by gathering stories through supportive supervision visits and online meetings.^24^ Three examples; establishing community funds to improve access to health centres among poor families, improving equitable water access, and tailored support for elderly people are shown in Box 1. While each addresses a different social determinant of health, these examples share common characteristics; they were primarily community-led, require collective action, and require trust-building and effective communication.

Box 1 **Examples of community-led social prescribing in Lao PDR.**

**Example 1: Establishing community funds to improve access to health centres among poor families in Sakuan village, Luang Prabang**

Sakuan village is an ethnic minority and mainly subsidence agriculture community in Luang Prabang province in northern Lao PDR. Its 900 residents experience challenges in accessing services due to the remote location, lack of transport, poverty, poor road conditions particularly during the rainy season, and extremely weak telecommunications connectivity. After CONNECT support, the village authorities established a revolving fund with fees collected from activities such as conflict mediation which could be used to support the cost of accessing services or other acute needs. This has led to an increase in essential service use such as antenatal care and delivery in health centres; for example, a mother who had given birth at home in the past described receiving a flexible loan of 700,000 LAK (35 USD) to cover costs and support to arrange transport to the hospital accompanied by the village health volunteer. While health staff were aware that many women gave birth at home, they had not managed to solve the issue simply by providing health education or counselling—it required a community-led solution to tackle access barriers.

**Example 2: Addressing inequitable water access in Nakuan village, Bolikhan district, Bolikhamsai**

In Nakuan village in remote Bolikhamsai, water access impacts the health and livelihoods of residents. A new gravity-fed water system was established by the government in 2014. However, by 2022, it was only reaching 30 percent of the households, predominantly those with wealth or connections with the village chief who had a high water usage. The direct water pipe connection to the headworks caused inequitable water access which remained unsolved for about 2 years. The district government and health centre staff were aware of the issue and had tried to negotiate with the village authorities and enforce regulations, but this approach only escalated conflict and tensions and did not succeed. In early 2024, CONNECT supported the village authorities for trust-building, two-way communication, games and scenario-based planning in order to identify affected families, and then visited the households that connected unauthorized pipes and invited them for discussion at the village office to share their ideas on how to ensure water access for everyone. Through involving the whole community and fostering respectful communication and mutual understanding, they reached an agreement for more equitable distribution by removing unauthorized pipes and support for poorer households. By July 2024, water access across the village had significantly improved.

**Example 3: Social support for elderly people in Phonxay village, Ngoi district, Luang Prabang**

Identifying vulnerable community members and developing community-led support mechanisms is a key part of the CONNECT approach. In June 2024, the CONNECT team together with village authorities found a 93 year old man living alone when conducting onsite supportive supervision in Phonxay health centre’s catchment area. He lived with only a bamboo shelter and vegetables he grew himself plus occasional rice donations, and was not registered as a village resident or in the poor family list. After talking to him to build trust and learn more about his situation, and providing him with some essentials, his case was raised during a meeting with the district and provincial authorities. Health providers acted by consulting with the village chief to register him on the list of poor families, which means he is eligible for a health insurance card for poor families and can access free health services. He also receives an annual fund of 700,000 kip ($35) from a hydropower company operating in the area, and support from village authorities when distributing rice or other goods.

## Lessons learned to guide social prescribing in low-resource settings

Community-led actions to address local SDH in the CONNECT Initiative show how non-formalised social prescribing can operate in a low resource setting without strong formal health and social systems. While the recent global definition of social prescribing emphasises that social prescribing may involve trusted individuals within communities,^3^ this co-production has been less captured within the evidence base from mainly high-income countries. However, reports from other LMICs where social prescribing has not been formally defined or integrated into systems demonstrate similar characteristics to Lao PDR. For example, in Indonesia, there are a number of grassroots initiatives which have shared values aligned with the concept, such as those which promote community empowerment through education and training, and community-based exercise for people with diverse health conditions which have shown positive social impact.^25^ The approach, outcomes and examples of community actions on the social determinants of health in the CONNECT Initiative can thus provide a number of insights into the formulation and adaption of social prescribing in LMICs.

First, in a setting with low access to health systems and limited capacities, social prescribing may not be conducted by formal health or social providers. CONNECT has strengthened and leveraged networks within the community to co-identify resources and solutions through inclusive and participatory discussions among community members. When basic infrastructure such as clean water is not in place, the issue is more structural and collective than individual. While identification of the needs of social prescribing reported from HICs mostly occurs during contact between individuals such as patients and healthcare providers during their visits, needs for structural support may be more easily and adequately identified in the community in LMICs. Health centres, as an integral part of the community, facilitate participatory discussions while providing technical support and health services to the community. Healthcare providers do not only identify and prescribe social interventions and support individuals as in high-income contexts,^26^ but also require additional skills in facilitating participatory processes to support the community to identify vulnerable populations and their needs. These processes are also supported by local authorities who have influence in the community, showing the need to develop facilitation skills in multi-sectoral local actors. In the examples from CONNECT, while the health staff and local authorities may be aware of the issue, it was community members who identified and led the development of a “prescription”, requesting their support as required.

Second, in the absence of formalized social mechanisms to address SDH, community-led social prescribing often requires collective action with collaboration from the whole community. CONNECT uses an asset-based approach aiming to find and build upon existing community potential in resource-limited settings. For villagers, meeting basic survival needs must usually take priority over additional activities. Therefore, building on existing structures and resources is a more feasible and sustainable method than introducing new programmes, along with fostering understanding of the underlying causes of inequity. Community structures in Lao PDR—which include formal and informal systems of village governance—and where mutual reliance is a necessary part of daily life are well suited to this approach which relies strongly on social capital.^27^^,^^28^ For example, in the case of village funds to support women accessing essential care, the village authorities identified an existing resource (fees collected for services such as conflict mediation) and organised to utilise them for the benefit of vulnerable community members. Where formal support mechanisms are available, it may require coordination between village authorities, health staff and local government to enable community members to connect with these. Examples include the elderly man who was supported to register for free health services, funds allocated to poor families, and community collaboration to provide food or other essentials.

Third, in the absence of existing social mechanisms to refer to, social prescriptions need to be co-developed within the community, which requires strong ownership and trust. Collected evidence from the CONNECT Initiative such as increased vaccination acceptance demonstrates how a sense of shared responsibility, understanding and leveraging existing structures, and identifying influential people in the community are all necessary to collectively respond to local challenges in an appropriate manner in Lao PDR.^22^^,^^29^ Additionally, the co-development of solutions may require awareness of and efforts to shift hierarchies within and between communities and local authorities. For example, in the case of improving water access, initial efforts by the district government and health centre staff failed when they tried to negotiate with the village authorities and enforce regulations, as this relied on external processes and did not consider the tensions and hierarchies present within the village. The CONNECT approach was applied to foster trust-building, respectful communication and shared understanding between those with different levels of power in order to find mutually beneficial solutions. Therefore, facilitators also require skills and awareness in understanding and addressing local power relations and their impact on health equity.

Implementing community-led social prescribing in LMICs may also face certain limitations and challenges. As we have shown, this approach appears to be successful within the rural community context of Lao PDR, a single party socialist state with relatively coherent governance structures from central to village level, and cultural emphasis on reciprocity. While it includes some similar characteristics to grassroots health initiatives in other LMICs, the CONNECT method has not yet been tested in other socio-political settings. Secondly, while community support is important for addressing local SDH within low-resource settings with limited service access, there is a risk that this approach may shift responsibility for service provision away from the state, providing an extra burden and expectation for residents to fulfil this responsibility. Third, it is important to be aware of power relations within communities, which are not homogenous and may include groups or individuals who experience social stigma or discrimination. These include ethnic or religious minorities, people living with HIV, sex workers, migrants, LGBTQ + youth and women-headed households, whose voices and needs are not always fairly represented within community decision-making. Equity-promoting policies, multisectoral collaboration including civil society and destigmatization training for local authorities and healthcare providers are therefore important to shift power relations more systematically. Finally, it is crucially important for community-led social prescribing to be “asset-based” through finding and utilising existing local resources in order to avoid reliance on external funding beyond the initial phase, otherwise the process may become unsustainable.

## Conclusion: beyond prescription to co-production?

Examples of community-led prescribing in Lao PDR supported by the CONNECT Initiative show potential for addressing SDH and improving access to healthcare for vulnerable groups. This approach is contextually appropriate for Lao PDR, with a mainly village-based population with low access to resources but relatively coherent local governance structures. This collective method of developing solutions also calls into question the concept of “prescribing” itself, which, in medical practice, describes a process by which a physician diagnoses and decides on a course of action for the patient. Instead, CONNECT facilitates the shared identification of needs and co-production of solutions, without necessarily relying on an authority decision-maker. Reframing this as community-led social prescribing—or co-production—may therefore assist in decolonizing health terminology, especially in non-Western contexts. While the approach might be suitable for LMICs with similar characteristics, there are also mutual learning opportunities for HICs wishing to establish community-led support services and to strengthen social cohesion. Furthermore, in the context of declining development aid and concerns about the ongoing sustainability of public health interventions,^30^ an asset-based approach which aims to build trust and facilitate locally-led collective action has the potential for long-term viability because it does not rely on externally prescribed solutions.

## Contributors

SK, EE conceptualized the article and wrote the original draft. PO, KP, SN led the intervention and reviewed the draft. OS, KV, ST, SM collected data, participated in the intervention and reviewed the draft. SB contributed to writing and revising the draft. YLP, TA reviewed and revised the draft.

## Data sharing statement

The data supporting the findings of this study are available within the article.

## Declaration of interests

There is no conflict of interest.
